# Respiratory Tract Infection: A Risk Factor for the Onset and Relapse of Adult-Onset Minimal Change Disease in Southern China

**DOI:** 10.1155/2018/1657208

**Published:** 2018-08-28

**Authors:** Huanqin Han, Shujun Wang, Yanting Liang, Jieping Lin, Lei Shi, Lin Ye, Shiting Song, Minjun He, Shihao Li, Futong Chen, Qingjun Pan, Hua-feng Liu

**Affiliations:** ^1^Key Laboratory of Prevention and Management of Chronic Kidney Disease of Zhanjiang City, Institute of Nephrology, Affiliated Hospital of Guangdong Medical University, Zhanjiang, Guangdong 524001, China; ^2^Department of Infectious Disease, Affiliated Hospital of Guangdong Medical University, Zhanjiang, Guangdong 524001, China; ^3^The First Clinical Medical College, Guangdong Medical University, Zhanjiang, Guangdong 524001, China

## Abstract

**Aims/Introduction:**

Steroid resistance and frequent relapse are problems in the treatment of minimal change disease (MCD). However, epidemiological factors that influence steroid-resistant and relapse of MCD are rarely reported. This study evaluated potential factors that influence the onset and relapse of MCD and the epidemiological features of southern Chinese patients with adult-onset MCD.

**Patients and Methods:**

Patients with adult-onset MCD were included from the Affiliated Hospital of Guangdong Medical University, which is located in the southernmost part of China's mainland, between 2015 and 2016. Potential influencing factors were investigated.

**Results:**

Eighty-seven patients with incipient MCD were enrolled, and 85 of these patients were followed up; 71.8% (61/85) were steroid-sensitive and 28.2% (24/85) were steroid-resistant. In terms of seasonal distribution, the highest rate of incipient cases was in spring (39.1%, 34/87), which also showed a high rate of relapse cases (29.7%, 22/74). Among patients who were followed up for more than half a year and whose proteinuria completely resolved (69.4%, 59/85), 52.5% (31/59) were without relapse and 47.5% (28/59) were with relapse. Patients without relapse were older than those with relapse (*P*<0.05). Before disease onset, 20.7% (18/87) of patients with incipient MCD were diagnosed with infection, including 94.5% (17/18) with respiratory tract infection. Fourteen patients in complete remission posttreatment developed an infection before relapse, including 85.7% (12/14) with respiratory tract infection.

**Conclusion:**

Steroid resistance and frequent relapse are current challenges for the treatment of adult-onset MCD in southern China, and respiratory tract infection may be a risk factor for onset and relapse. Additionally, younger patients with MCD tend to have more frequent relapse.

## 1. Introduction

Minimal change disease (MCD) is a glomerular disease clinically manifested as nephrotic syndrome (NS); the primary characteristics of MCD are no obvious pathological changes on light microscopy and extensive disappearance of podocyte foot processes on electron microscopy. Zhou et al. [1] reported that MCD accounts for 6.3%-13.4% of glomerular diseases in Chinese patients older than 14 years old. Moreover, the morbidity rate of MCD increases yearly [1]. Though patients with MCD can easily achieve remission, the relapse rate is high, and MCD can transform into focal segmental glomerulosclerosis (FSGS) or even end-stage renal disease (ESRD) [2–6].

The pathogenesis of MCD is not yet well known. Infection is one factor that is thought to potentially influence the rate of relapse of MCD; it is also the most common complication [7]. In addition, it was reported that the onset of NS in children and adolescent has a seasonal distribution [8], while the influence of seasonality on adult cases, especially adult-onset MCD, has not been reported. Most cases of MCD occur in children, and the current understanding and research of the disease are based on data derived from children. However, the incidence rate and clinical features of NS are variable according to patient age, sex, location, and race [8–12]. Moreover, factors that influence the relapse rate of MCD and the epidemiological features of adult-onset MCD are rarely reported [4, 5, 13].

In this study, patients with adult-onset MCD were included and followed up in order to investigate factors that influence the relapse rate of MCD and the epidemiological features of MCD in southern Chinese adults.

## 2. Materials and Methods

### 2.1. Patient Recruitment

This study included hospital inpatients with idiopathic MCD who were admitted to Affiliated Hospital of Guangdong Medical University, which is located in the southernmost part of China's mainland, from January 2015 to December 2016. All patients were older than 16 years old, were diagnosed with idiopathic MCD confirmed by clinical and renal pathological findings, and met the diagnostic criteria of NS. Patients with secondary MCD or idiopathic MCD combined with acute renal injury were excluded.

### 2.2. Therapeutic Regimen

The basic therapeutic regimen was oral prednisone 1 mg/kg/d; then, the dose was gradually reduced by 10% every 2 weeks. The total therapeutic course was approximately 9-12 months. An equal dose of methylprednisolone could be used in patients with hepatic dysfunction or hepatitis B virus infection. Corticosteroid combined with an immunosuppressant, such as cyclophosphamide, could be used in patients with steroid resistance or frequent relapse.

### 2.3. Study Methods

Epidemiological and clinical data of the included patients were followed up and recorded, including age, sex, therapeutic regimen, complete remission rate, time required to achieve remission, relapse rate of patients followed up for more than half a year or one year after urine protein became negative, seasonal distribution of patients with incipient and relapsed MCD, and the rate of infections before onset or during corticosteroid therapy. All data were entered into follow-up software (Siyuan Hospital Customer Relationship Management, Siyuan Technology Industrial Co., Ltd, Changsha, China) of our hospital. Potential factors (age, sex, infection) affecting primary steroid response and potential factors (age, sex, steroid sensitivity, time required to achieve remission during primary therapy) affecting relapse were analyzed, and cases with different outcomes were compared between groups.

### 2.4. Serum Levels of IgE Detection

Serum levels of IgE were tested with a commercial Human IgE ELISA Kit (ab108650) (Abcam, Cambridge, MA, USA). Healthy controls with no differences in age, sex, or race to idiopathic MCD patients were enrolled into the present study.

### 2.5. Definition

Response to steroid therapy was defined as follows: (1) steroid-sensitive: within 8 weeks after steroid therapy, urine protein became negative (negative twice not in the same day, or 24-h urine protein <0.3 g); (2) steroid-resistant: NS not achieving remission after steroid therapy for 8 weeks; (3) relapse: relapse after urine protein became negative, with urine protein ≥1+; (4) frequent relapse: ≥2 episodes of relapses in 6 months or ≥3 episodes of relapse in 1 year [5, 14].

### 2.6. Ethical Approval

This trial was registered with http://www.chictr.org.cn (registration number: ChiCTR-TRC-13003783) and approved by the Ethics Committee of Affiliated Hospital of Guangdong Medical University (No. PJ2017054). Consent was obtained from each participant.

### 2.7. Statistical Analysis

Data were analyzed using SPSS Statistics (version 23.0; IBM, USA). Data are presented as mean ± standard error or percentages. Differences were examined using Student's unpaired* t*-test for parametric data. Categorical data were examined using the chi-square test. The *P* value was considered statistically significant if less than 0.05.

## 3. Results

### 3.1. Patient Inclusion and Outcome

**Figure 1:**
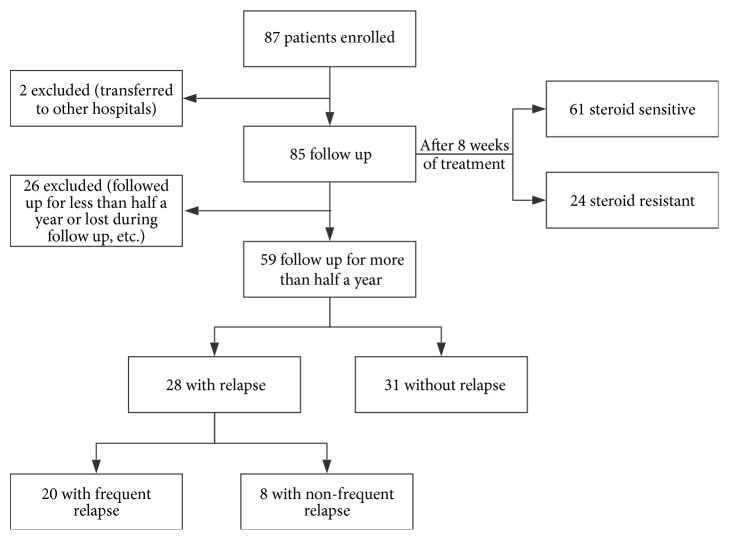
Flow chart of patients with minimal change disease included in the study.

Figure 1 shows the flow chart of patient inclusion and outcomes, including 87 patients with incipient MCD, consisting of 75 males and 12 females ranging from 16 to 66 years old. Two patients who were transferred to other hospitals were excluded; thus 85 patients were followed up. After 8 weeks of treatment, 61 patients were steroid-sensitive and the steroid-sensitive rate (complete remission) was 71.8% (61/85) with initial therapy; 28.2% (24/85) were steroid-resistant. Patients who were followed up for less than half a year or lost during follow-up (20 patients), those who were medication noncompliant (5 patients), or those who developed spontaneous remission (1 patient) were excluded. Fifty-nine patients were followed up for more than half a year after urine protein became negative, including 52.5% (31/59) without relapse and 47.5% (28/59) with relapse (20 with frequent relapse). Of the 59 patients, 44 were followed up for more than 1 year after urine protein became negative, including 38.6% (17/44) without relapse and 61.4% (27/44) with relapse (19 with frequent relapse).

### 3.2. Seasonal Distribution of Incipient MCD

**Figure 2:**
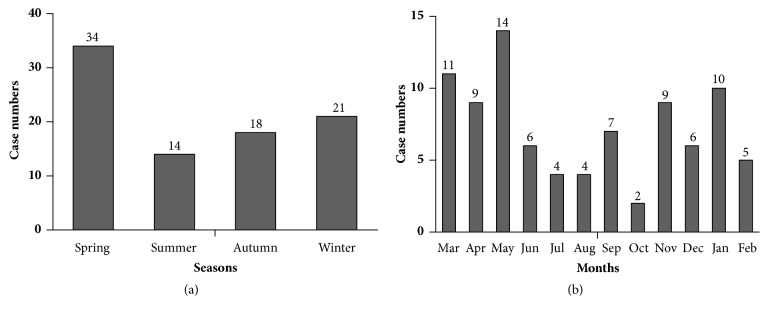
Distribution of incipient minimal change disease (MCD) between 2015 and 2016. (a) Seasonal distribution of patients with incipient MCD between 2015 and 2016. (b) Monthly distribution of patients with incipient MCD between 2015 and 2016.

Figure 2 shows the seasonal distribution of incipient MCD. Incipient MCD was diagnosed in 39.1% (34/87) of patients in spring, 24.1% (21/87) in winter, 20.7% (18/87) in autumn, and 16.1% (14/87) in summer. May had the most number of patients with initial onset of MCD (13, 16.1%) and October had the least (2, 2.3%).

The relative proportion of incipient MCD (MCD case numbers divided among all admitted case numbers in the hospital) was defined due to the variability of incipient MCD cases by month, with the lowest proportion being in February, which includes Spring Festival in China, and the highest proportion in August. Results showed that the relative proportion in spring, winter, autumn, and summer gradually decreased (*P* = 0.016). Paired comparison showed that the relative proportion in spring was larger than that in summer (*P* = 0.004), autumn (*P* = 0.025), and winter (*P* = 0.158,). May was the month with the highest relative proportions (Table 1).

### 3.3. Seasonal Distributions of MCD Relapse

**Figure 3:**
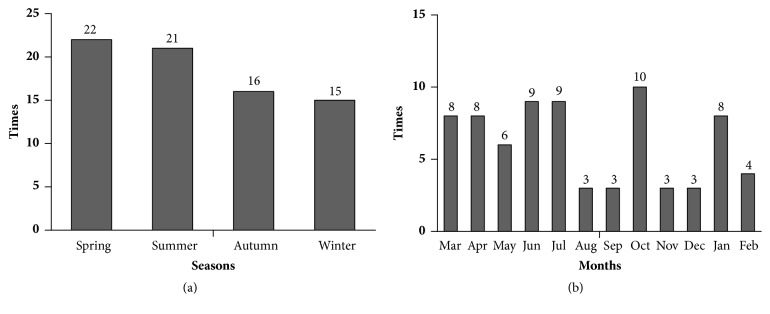
Distribution of minimal change disease (MCD) relapses between 2015 and 2016. (a) Seasonal distribution of MCD relapse between 2015 and 2016. (b) Monthly distribution of MCD relapse between 2015 and 2016.

Fifty-nine patients with incipient MCD were followed up for more than half a year from January 2015 to June 2017. Seventy-four episodes of relapse occurred, including 29.7% (22/74) in spring, 28.3% (21/74) in summer, 21.6% (16/74) in autumn, and 20.3% (15/74) in winter. The majority of relapses occurred in spring, similar to the distribution of MCD onset.  Figure 3 shows the seasonal distribution of relapsed MCD.

### 3.4. MCD and Infection

From January 2015 to June 2017, 20.7% (18/87) of patients with MCD had infection before disease onset, including 94.5% (17/18) with respiratory tract infection (including upper respiratory tract infection, bronchitis, and pulmonary infection). After complete remission and drug withdrawal, patients had infection for 14 times before relapse, including 85.7% (12/14) with respiratory tract infection. During corticosteroid therapy, 70 episodes of infection occurred; pulmonary infection (28.6%, 20/70) was most common. No patient died due to infection during follow-up. Infection sites and case numbers are reported in Table 2.

### 3.5. Influence of Sex, Age, and Infection before Initial Therapy on Response to Treatment

In this study, 85 cases of incipient MCD were treated with corticosteroids. Patients were divided into steroid-sensitive and steroid-resistant groups according to the response to steroid; there was no significant difference between groups in terms of sex, age, or occurrence of infection before initial therapy (all* P* > 0.05) (Table 3).

### 3.6. Influence of Sex, Age, and Time to Initial Remission on Relapse

After urine protein became negative, 59 patients with MCD were followed up regularly for more than half a year. Among these patients, age ranged from 18 to 49 years old, and the average follow-up duration was 55.1 ± 32.9 weeks.  Table 4 reports correlations of sex, age, steroid sensitivity at initial therapy, and time required to achieve remission after initial therapy with later relapse; results suggest that the nonrelapse group included more patients of older age than the relapse group (*P *< 0.05). No significant difference was observed between sex, steroid sensitivity, and time required to achieve remission after initial therapy (all* P *> 0.05).

For 28 patients in the relapse group, 20 cases showed frequent relapse and 8 cases showed infrequent relapse, with mean ages of 18.5 ± 4.3 and 19.5 ± 3.9 years old, respectively. The mean age of the infrequent relapse group was slightly older than that of the frequent relapse group, although this was not statistically significant (*t* = 0.575,* P* = 0.570).

### 3.7. Serum Levels of IgE in Incipient MCD

The results showed that serum IgE levels in patients with MCD (371.8 ± 248.5 IU/ml) (n = 87) were significantly higher than in the normal control group (47.2 ± 31.6 IU/ml) (n = 36; 31 male, 5 females; Age: 21.8 ± 13.6 years) (*P* < 0.05).

## 4. Discussion

The incidence rate and clinical features of NS are recognized to be variable according to patient age, sex, location, and race [8–12]. In the current study, patients were from the Affiliated Hospital of Guangdong Medical University, which is located in the southernmost part of China's mainland, a region with a tropical and subtropical monsoon climate and hot and humid weather. The patients included in this study were diagnosed with adult-onset MCD.

Our study suggests that most patients with adult-onset MCD show good response to corticosteroid treatment, in agreement with reports from other regions [3, 4]. The relapse rate of adult MCD after remission may be lower than that of children, but there is a lack of high-quality medical evidence. In this study, after urine protein became negative, 59 patients were followed up for more than half a year with a relapse rate of 47.5%; the relapse rate increased to 61.4% with one year follow-up. Compared to the typical and high-quality research data concerning relapse of children with MCD conducted by Tarshish et al. [15], it seems that long-term remission in adult patients was not higher than that of children.

We analyzed the seasonal features of incipient and relapsed MCD to investigate the external factors associated with MCD onset in this patient group. We found that incipient MCD shows seasonal differences; the incidence rate is highest in the spring, followed by winter. Similarly, Chang et al. [8] analyzed 4083 cases of primary NS in children and adolescence in Taiwan and found that incipient diseases occurred most commonly in winter, followed by spring. In fact, most cases of MCD relapse also occurred in the spring, though there was no significant difference among seasons. In our opinion, the difference in seasonality between incipient and relapse MCD may be due to many factors. Not only season, but also climate, individual immunity, patient compliance, and infection rate may influence the relapse trends.

As adult MCD shows obvious seasonal differences, we assumed that the high incidence rates of MCD in spring and winter may be due to high rates of respiratory tract infection during these two seasons, and upper respiratory tract infection may be an important cause of MCD onset. Second, MCD may be a disease related to allergy, and its activity is associated with increased serum IgE levels [16–19], an antibody that mediates allergy. In fact, in this study, we also found that serum IgE levels in patients with MCD were significantly higher than in the normal control group. Additionally, spring is the season with the high rates of allergic diseases, such as allergic rhinitis and bronchial asthma [20]. Thus, the high rates of MCD in the spring and winter in the current study further suggest that MCD may be a disease associated with infection and allergy. This phenomenon also suggests that we should include analysis of allergic factors, such as IgE, basophils, and eosinophils, when studying the pathogenesis of MCD.

Infection is one of the main causes of relapse and poor treatment efficacy in patients with NS. MCD, which presents as NS, may be complicated by infection because of obviously lower IgG levels, immune dysfunction [21], subcutaneous effusion [22], and use of corticosteroids. However, these data are mainly acquired from children. Our study indicates that incipient and relapsed adult MCD are usually associated with previous infection, especially respiratory tract infection. More importantly, many patients have edema and massive proteinuria following respiratory tract infection; it is presumed that infection is not only a complication of MCD, but also a reason for the development of incipient or relapsed MCD. Thus it would be very beneficial for patients with MCD to avoid respiratory tract infection.

Up to now, studies on the response of NS to steroids and the NS relapse rate have primarily been performed in children, with very few investigations of adults, and extremely rare studies enrolling Chinese adults. However, it is known that predicting steroid response and relapse in patients with MCD is of concern. Here, we analyzed the basic information of patients in steroid-sensitive and steroid-resistant groups, but did not identify any influence of sex, age, or initial therapy on steroid sensitivity. We also analyzed factors influencing MCD relapse in adult patients, but again did not find an effect of sex, steroid sensitivity at initial therapy, or time required to achieve remission after initial therapy. However, age was significantly association with relapse, as younger adult patients tended to show relapse or frequent relapse, but older patients tended not to relapse. These findings, based on our large patient sample size, were similar to another reported by Tse et al. [23]

## 5. Conclusions

Steroid resistance and frequent relapse are current challenges for the treatment of adult-onset MCD in southern China, and respiratory tract infection may be a risk factor for the onset and relapse of MCD in these patients. Younger patients with MCD are more likely to relapse or show frequent relapse.

## Figures and Tables

**Table 1:** Relative proportions of patients with incipient minimal change disease during each month and season.

Month	Incipient MCD (*n*)	All admitted patients (*n*)	Relative proportions (‰)	Season	Incipient MCD (*n*)	All admitted patients (*n*)	Relative proportions (‰)
Mar	11	13088	0.84	Spring	34	39096	0.87
Apr	9	12707	0.71				
May	14	13301	1.05				
Jun	6	12486	0.48	Summer	14	39261	0.36
Jul	4	13322	0.30				
Aug	4	13453	0.29				
Sep	7	12012	0.58	Autumn	17	37641	0.48
Oct	2	12584	0.16				
Nov	9	13045	0.69				
Dec	6	12781	0.47	Winter	21	35651	0.59
Jan	10	12735	0.79				
Feb	5	10135	0.49				

MCD, minimal change disease.

**Table 2:** Patients with minimal change disease with infection before onset or during corticosteroid treatment.

Infections	Before onset		Before relapse		During corticosteroid therapy		Total	
	Cases (*n*)	Proportions (%)	Times (*n*)	Proportions (%)	Times (*n*)	Proportions (%)	Times (*n*)	Proportions (%)
Upper respiratory tract infection or bronchitis	11	61.1	9	64.3	11	15.7	31	30.4
Pulmonary infection	6	33.3	3	21.4	20	28.6	29	28.4
Intestinal infection	0	0	1	7.1	12	17.1	13	12.8
Cutaneous or subcutaneous infection	0	0	0	0	12	17.1	12	11.8
Urinary infection	0	0	1	7.1	8	11.4	9	8.8
Spontaneous bacterial peritonitis	1	5.6	0	0	5	7.1	6	5.9
Septicemia	0	0	0	0	2	2.9	2	2.0

**Table 3:** Comparisons of sex, age, and infection before initial therapy between the steroid sensitive group and resistant group.

Characteristic	Steroid sensitive (*n* = 61)	Steroid resistant (*n* = 24)	*χ* ^2^/*t* value	*P *value
Male (n, %)	54 (88.5)	20 (83.3)	0.080	0.777
Age ($\overset{-}{x}\pm s$, years)	24.4 ± 15.9	22.4 ± 10.2	0.587	0.558
With infection before onset (n, %)	15 (24.6)	3 (15.0)	1.508	0.219

**Table 4:** Comparisons of sex, age, steroid sensitivity, and time to complete remission after initial therapy between the non-relapse group and relapse group.

Characteristic	Non-relapsers (*n* = 31)	Relapsers (*n* = 28)	*χ* ^2^/*t* value	*P *value
Male (n, %)	27 (87.1)	25 (89.3)	0.000	1.000
Age ($\overset{-}{x}\pm s$, years)	22.9 ± 9.4	18.8 ± 4.1	2.183	0.035
Steroid sensitive (n, %)	26 (83.9)	23 (82.1)	0.000	1.000
Time required to achieve remission ($\overset{-}{x}\pm s$, w)	5.7±4.8	5.1±4.4	0.532	0.597

## Data Availability

The data used to support the findings of this study are available from the corresponding author upon request.
